# The Involvement of Glucose in Hydrogen Gas-Medicated Adventitious Rooting in Cucumber

**DOI:** 10.3390/plants10091937

**Published:** 2021-09-17

**Authors:** Zongxi Zhao, Changxia Li, Huwei Liu, Jingjing Yang, Panpan Huang, Weibiao Liao

**Affiliations:** College of Horticulture, Gansu Agricultural University, 1 Yinmen Village, Anning District, Lanzhou 730070, China; zhaozongxi2021@163.com (Z.Z.); licxgsau5@163.com (C.L.); Ihwgsauedu@163.com (H.L.); yangjingjing202108@163.com (J.Y.); huangpp2243739575@163.com (P.H.)

**Keywords:** glucosamine, sucrose, starch, gene expression, sugar metabolism

## Abstract

Hydrogen gas (H_2_) and glucose (Glc) have been reported as novel antioxidants and signal molecules involved in multiple biological processes in plants. However, the physiological roles and relationships of H_2_ and Glc in adventitious rooting are less clear. Here, we showed that the effects of different concentrations Glc (0, 0.01, 0.05, 0.10, 0.50 and 1.00 mM) on adventitious rooting in cucumber were dose-dependent, with a maximal biological response at 0.10 mM. While, the positive roles of hydrogen rich water (HRW, a H_2_ donor)-regulated adventitious rooting were blocked by a specific Glc inhibitor glucosamine (GlcN), suggesting that Glc might be responsible for H_2_-regulated adventitious root development. HRW increased glucose, sucrose, starch and total sugar contents. Glucose-6-phosphate (G6P), fructose-6-phosphate (F6P) and glucose-1-phosphate (G1P) contents were also increased by HRW. Meanwhile, the activities of sucrose-related enzymes incorporating sucrose synthase (SS) and sucrose phosphate synthase (SPS) and glucose-related enzymes including hexokinase (HK), pyruvate kinase (PK) and adenosine 5′-diphosphate pyrophosphorylase (AGPase) were increased by HRW. Moreover, HRW upregulated the expression levels of sucrose or glucose metabolism-related genes including *CsSuSy1*, *CsSuSy6*, *CsHK1*, *CsHK3*, *CsUDP1*, *CsUDP1-like*, *CsG6P1* and *CsG6P1-like*. However, these positive roles were all inhibited by GlcN. Together, H_2_ might regulate adventitious rooting by promoting glucose metabolism.

## 1. Introduction

Adventitious roots (AR) are postembryonic roots which originate from the stem, leaf petiole and non-pericycle tissue of old roots [1]. Normally, inappropriate conditions including injury and stress promote AR formation. Recent results revealed that AR formation was positively regulated by plant hormones and signaling molecules, such as abscisic acid (ABA) [2], auxin [3], brassinolide (BR) [4], gibberellin (GA) [5], ethylene [6], hydrogen peroxide (H_2_O_2_) [7], nitric oxide (NO) [8], carbon monoxide (CO) [9] and hydrogen sulfide (H_2_S) [10] Additionally, molecular evidence illustrated that auxin- and ethylene-related genes and proteins are closely associated with the initiation and development of AR [11]. The complex responses regulated by these hormones, signaling molecules, genes and proteins are most likely to be achieved by a more detailed AR signaling process. However, whether there are some other novel singling molecule(s) involved in AR formation remains to be studied.

Hydrogen gas (H_2_), colorless, tasteless and flammable, is the structurally simplest gas in nature. Recently, research on H_2_ has progressed from focusing on its role as a fuel to its role as matter that is able to regulate multiple biological functions in animals and plants. Treatment with H_2_ relieved brain damage and inflammation after traumatic brain injury in rats [12]. At the same time, it was reported that hydrogen therapy was a potential and effective treatment for exercise-induced injury in sports medicine [13]. In plants, H_2_ is considered as an important signaling modulator that functions in plant responses against salt, heavy metals, low temperatures and paraquat stresses [14]. H_2_ actively controls a series of plant growth and development stages including seed germination, seedling growth, adventitious rooting and root elongation [14]. In addition, hydrogen rich water (HRW) could defer postharvest ripening and aging of kiwifruit [15]. Interestingly, H_2_ can interact directly with specific signaling pathways, including NO, CO and ethylene [16].

Starch is a ubiquitous storage polysaccharide in the plant kingdom. Adenosine 5′-diphosphate pyrophosphorylase (AGPase) is a key enzyme governing starch synthesis. In the process of plant metabolism, starch is hydrolyzed into monosaccharides such as glucose (Glc). Glc is an important member of monosaccharides which exists widely in animals and plants. Simultaneously, Glc is recognized as a central signaling molecule that balances the requirement of nutrient and energy in plants. Hexokinase (HK), as an enzyme in glycolysis, phosphorylates glucose to glucose-6-phosphate [17]. Both HK-dependent and HK-independent glucose signal transduction pathways appear to coexist in plants [18]. Sucrose is synthesized using cytosolic phosphotriose as a substrate. Sucrose may then be transported to sink organs or it is cleaved by invertase to Glc and fructose [19]. Fructose-6-phosphate (F6P) and sucrose phosphate synthase (SPS) have been recognized as the rate-limiting products or enzymes in the process of sucrose synthesis [20]. Glc acts as a primary signal molecule in plant response against drought stress and heat stress [21]. Simultaneously, it was reported that Glc regulated a series of growth and development stages, including seed germination, seedling development, embryo development, cell division, stomatal movement, seed dormancy, leaf senescence and fruit ripening [22,23]. Previous results also elaborated that Glc significantly regulated AR development in *Arabidopsis thaliana* [24].

Previous studies have shown that both H_2_ and Glc regulate AR development in plants. However, there is no information regarding the crosstalk between H_2_ and Glc in adventitious rooting. The purpose of the research study was to investigate the roles and interaction of H_2_ and Glc in AR development. Deeper insights into the interplay of various signaling molecules with H_2_ will help provide a road-map for H_2_-regulated AR development.

## 2. Results

### 2.1. Effect of Different Concentrations Glc on AR Development

When compared with the control, 0.01 mM Glc had no significant effect on root number (Table 1). However, treatments with 0.05, 0.10 and 0.50 mM Glc significantly increased root number (Table 1). Root length was higher in 0.01, 0.05, 0.10 and 0.50 mM Glc treatments than in the control (Table 1). Compared to the control, 1.00 mM Glc significantly inhibited both root number and root length. The results indicated that Glc treatment affected AR development in a dose-dependent manner. Among the different concentrations, the maximum root number and root length were observed at 0.10 mM concentration of Glc (Table 1). Thus, 0.10 mM Glc was used as a treatment for further studies during the rooting process.

### 2.2. Involvement of Glc in HRW-Regulated AR Development

**Figure 1:**
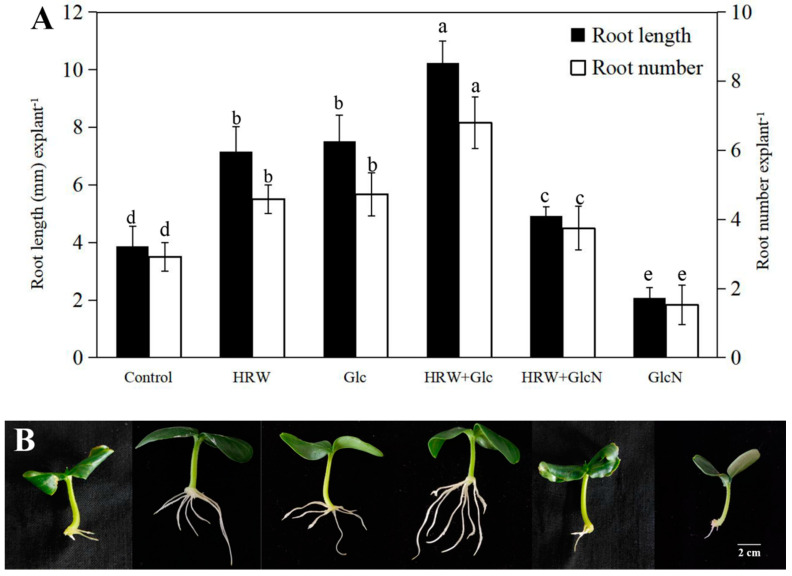
Effects of HRW, Glc and GlcN on root number and root length (**A**) and phenotype (**B**) of cucumber explants treated with 50% HRW, 0.10 mM Glc and 0.10 mM GlcN alone or together. n = 30 explants per replicate. Bars with different lower case letters were significantly different (*p* < 0.5). HRW: hydrogen rich water; Glc: glucose; GlcN: glucosamine.

Our previous study found that HRW treatment could promote the formation of AR in cucumber, and the optimal concentration was 50% [25], which were also used in this study. To analyze the roles of Glc in H_2_-regulated AR development, cucumber explants were treated with 50% HRW, 0.10 mM Glc and 0.10 mM Glc inhibitor glucosamine (GlcN) alone or together (Figure 1). Cucumber explants treated with 50% HRW or 0.10 mM Glc exhibited significant increase in root number and root length (Figure 1). Additionally, co-treatment with HRW and Glc significantly enhanced adventitious rooting in comparison with HRW or Glc treatment alone. When GlcN was added to HRW, the positive effects of HRW on rooting was weakened. GlcN treatment alone significantly inhibited adventitious rooting in comparison with the control (Figure 1). These results indicated that Glc might be involved in H_2_-promoted AR development in cucumber.

### 2.3. Effects of HRW, Glc and GlcN on Glucose, Sucrose, Starch and Total Sugar Contents during Adventitious Rooting 

**Figure 2:**
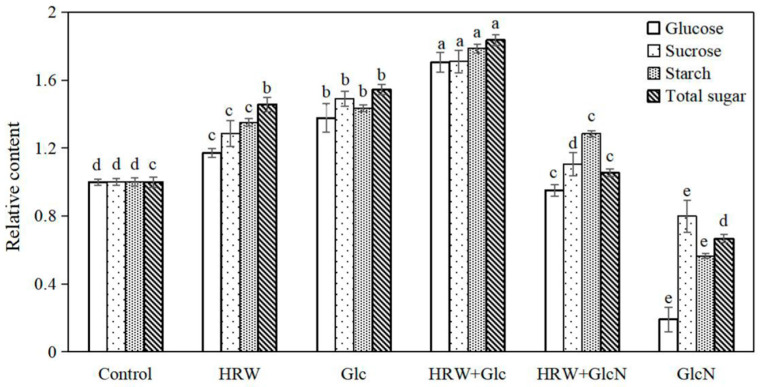
Effects of HRW, Glc and GlcN on glucose, sucrose, starch, and total sugar contents during adventitious rooting. n = 30 explants per replicate. Bars with different lower case letters were significantly different (*p* < 0.5). HRW: hydrogen rich water; Glc: glucose; GlcN glucosamine. The relative content (compared to the control) is displayed in the figure.

Compared with the control, HRW and Glc treatments significantly increased the contents of glucose, sucrose, starch and total sugar (Figure 2). Additionally, glucose, sucrose, starch and total sugar contents in explants treated with HRW plus Glc were significantly increased compared to the HRW or Glc alone treatments. The glucose, sucrose, starch and total sugar contents in Glc inhibitor GlcN treatment were lower than that in the control (Figure 2). 

### 2.4. Effects of HRW, Glc and GlcN on Hexose Phosphate Content during Adventitious Rooting

**Figure 3:**
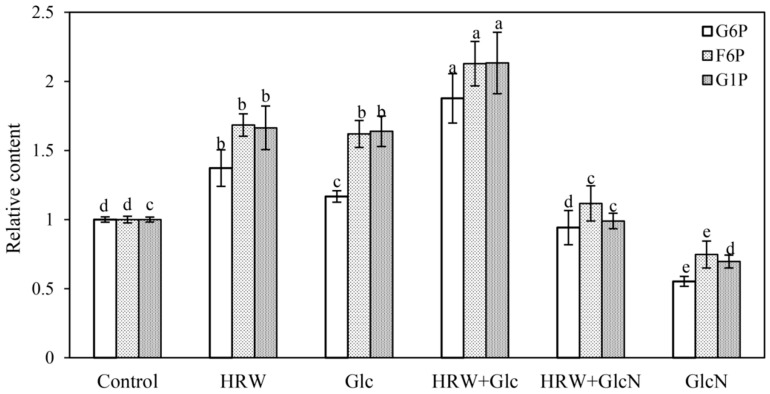
Changes in the contents of G6P, F6P and G1P of cucumber explants under the treatments of HRW, Glc and GlcN. n = 30 explants per replicate. Bars with different lower case letters were significantly different (*p* < 0.5). HRW: hydrogen rich water; Glc: glucose; GlcN: glucosamine; G6P: glucose-6-phosphate; F6P: fructose-6-phosphate; G1P: glucose-1-phosphate. The relative content (compared to the control) is displayed in the figure.

As shown in Figure 3, HRW and Glc treatments significantly increased G6P, F6P and G1P contents compared with the control. In comparison with the separate treatment of HRW and Glc, HRW + Glc treatment significantly increased the contents of G6P, F6P and G1P. However, when GlcN was added, the positive impact of HRW on the G6P, F6P and G1P contents declined significantly (Figure 3). Compared with the control, GlcN alone treatment significantly decreased the contents of G6P, F6P and G1P (Figure 3). 

### 2.5. Effects of HRW, Glc and GlcN on Key Enzymes of Glucose Metabolism during Adventitious Rooting

**Figure 4:**
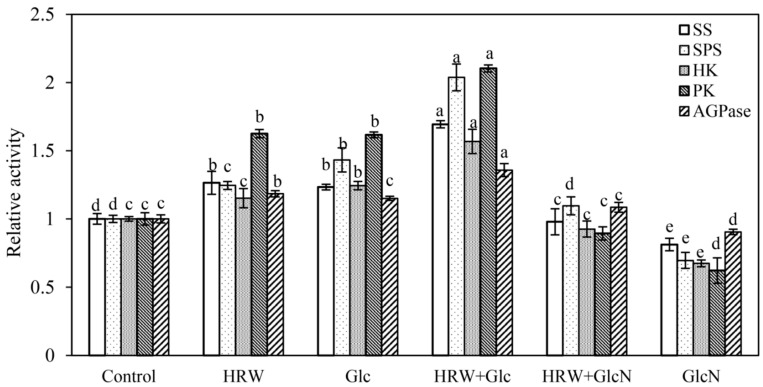
Effects of HRW, Glc and GlcN on SS, SPS, HK, PK and AGPase enzyme activities of cucumber explants. n = 30 explants per replicate. Bars with different lower case letters were significantly different (*p* < 0.5). HRW: hydrogen rich water; Glc: glucose; GlcN: glucosamine; SS: sucrose synthase; SPS: sucrose phosphate synthase; HK: hexokinase; PK: pyruvate kinase. AGPase: adenosine 5′-diphosphate pyrophosphorylase. The relative activity (compared to the control) is displayed in the figure.

Compared to the control, the activities of SS, SPS, HK, PK and AGPase were significantly enhanced by Glc or HRW (Figure 4). Meanwhile, HRW plus Glc treatment showed higher SS, SPS, HK, PK and AGPase activities than HRW or Glc treatment (Figure 4). Conversely, GlcN significantly reduced the enhancement caused by HRW. The activities of SS, SPS, HK, PK and AGPase in GlcN treatment alone were lower than that in the control (Figure 4). 

### 2.6. Effects of HRW, Glc and GlcN on the Expression Levels of CsSuSy1, CsSuSy6, CsHK1, CsHK3, CsUDP1, CsUDP1-Like, CsG6P1 and CsG6P1-Like Genes during Adventitious Rooting

**Figure 5:**
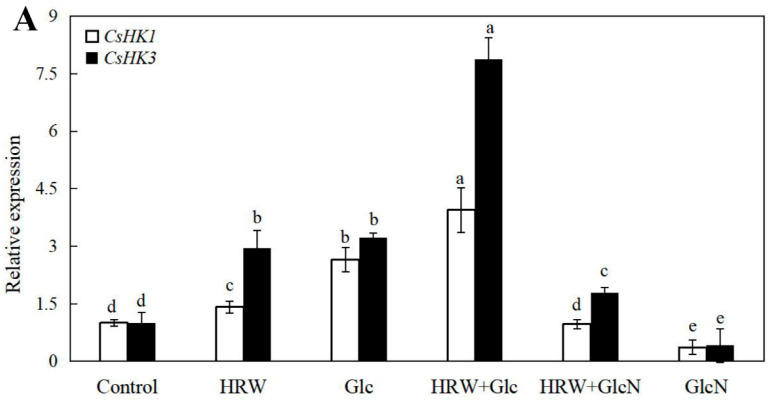

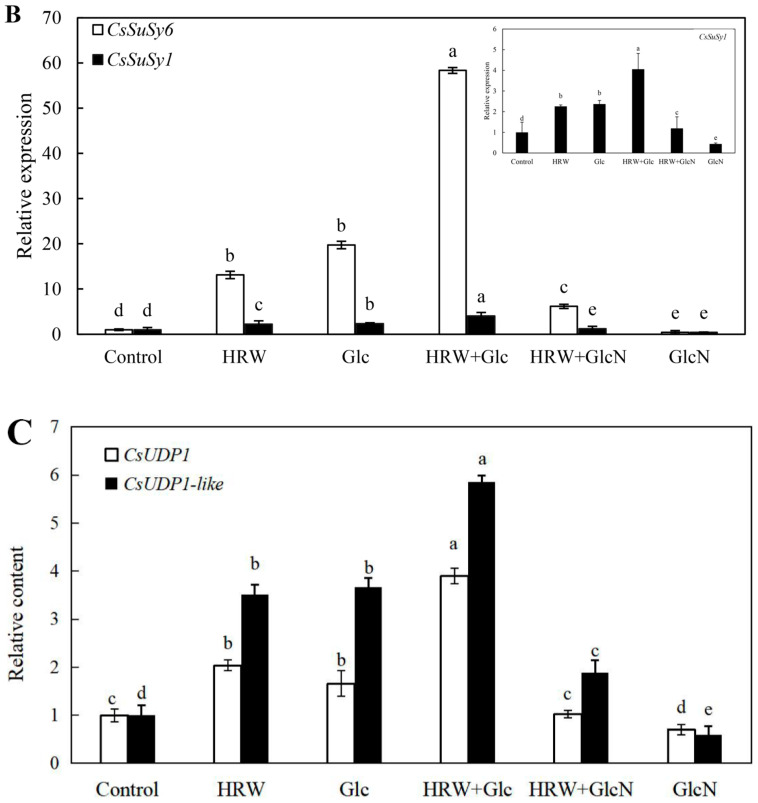

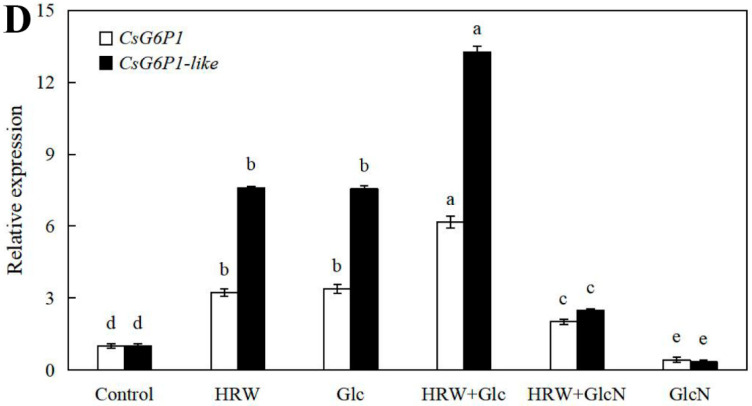
Effects of HRW, Glc and GlcN on the expression levels of *CsSuSy1* and *CsSuSy6* (**A**), *CsHK1* and *CsHK3* (**B**), *CsUDP1* and *CsUDP1-like* (**C**), *CsG6P1* and *CsG6P1-like* (**D**) genes in cucumber explants. n = 30 explants per replicate. Bars with different lower case letters were significantly different (*p* < 0.5). HRW: hydrogen rich water; Glc: glucose; GlcN: glucosamine.

As shown in Figure 5, the expression levels of *CsSuSy1*, *CsSuSy6*, *CsHK1*, *CsHK3*, *CsUDP1*, *CsUDP1-like*, *CsG6P1* and *CsG6P1-like* genes in Glc or HRW treatments were significantly upregulated in comparison with the control. Additionally, the expression levels of *CsSuSy1*, *CsSuSy6*, *CsHK1*, *CsHK3*, *CsUDP1*, *CsUDP1-like*, *CsG6P1* and *CsG6P1-like* genes were the highest in HRW plus Glc treatment (Figure 5). Conversely, the expression levels of *CsSuSy1*, *CsSuSy6*, *CsHK1*, *CsHK3*, *CsUDP1*, *CsUDP1-like*, *CsG6P1* and *CsG6P1-like* genes were significantly reduced when GlcN was added (Figure 5).

## 3. Discussion

Generally, AR plays a vital role in nutrient and water absorption. Their formation is widely used for plant clonal propagation. Previous results in our lab have shown that H_2_ as a positive regulator regulated adventitious rooting in cucumber [26]. However, there is little research in the crosstalk between H_2_ and Glc during adventitious rooting. Here, we focus on the involvement of Glc in H_2_-regulated AR formation.

Glc as signaling molecule has been the concern of by many researchers. So far, accumulating evidence indicated that Glc participated in the regulation of various growth and development, such as seed germination, seedling development and fruit ripening [15]. In the present study, we illustrated that Glc treatment promoted adventitious rooting in cucumber explants in a dose-dependent manner, and 0.10 mM Glc treatment was the most effective concentration (Table 1). Singh [27] reported that Glc controlled seedling root growth direction through regulating root waving and coiling in *Arabidopsis thaliana*, leading to altered root architecture. Mishra [24] also found that increasing Glc concentration increased root length, number of lateral roots and root hairs in *Arabidopsis thaliana* seedlings. Therefore, Glc plays a principal role in the regulation of root growth. A note of caution is that the effect of anaerobic bacteria on which glucose promoted adventitious rooting of cucumber has not been ruled out. Additionally, Zhu [16] found that NO was involved in H_2_-regulated adventitious rooting of cucumber. Chen [9] reported that CO was involved in H_2_-regulated adventitious rooting in cucumber under water stress. For the first time, our results indicated that Glc might be involved in H_2_-regulated AR development in cucumber (Figure 1). Therefore, signal molecules including nitric oxide (NO), carbon monoxide (CO) and Glc all were required for H_2_-regulated AR formation in cucumber.

In this study, we demonstrated, for the first time, that HRW treatment significantly increased the contents of glucose, sucrose, starch and total sugar during AR formation. However, the positive effects of HRW were blocked by GlcN (an inhibitor of Glu), suggesting that H_2_ might regulate adventitious rooting by increasing glucose, sucrose, starch and total sugar contents (Figure 2). However, the effect of H_2_ on sugar metabolism in plants has not yet been demonstrated so far. In animals, Kim and Kim [28] reported that application of HRW could improve blood glucose control for insulin deficiency and insulin resistance. HRW also has beneficial effects on lipid and glucose metabolism in humans [29]. Additionally, CH_4_-medicated starch and sucrose metabolism regulated bulblet formation in *Lilium davidii* var. *unicolor* [30]. Therefore, studies on H_2_ regulating glucose metabolism in plants need to be further explored. Glycolysis is a cytoplasmic pathway which breaks down glucose into G6P or F6P [17]. Glucose is trapped by phosphorylation, with the help of the enzyme hexokinase [17]. Our result found that Glc significantly increased G6P, F6P and G1P contents during adventitious rooting (Figure 3). Glc has also been shown to modulate hypocotyl directional growth in *A. thaliana* [27]. Here, we found that HRW also enhanced AR formation via increasing G6P, F6P and G1P contents. However, the positive role was inhibited by GlcN (Figure 3). Thus, H_2_ regulated adventitious rooting by increasing Glu, G6P, F6P and G1P contents. 

Shi [31] revealed that SPS and SS were the key enzymes in sucrose accumulation in longan fruit. HK and PK played an essential role in Glc and fructose metabolism pathway [32]. Glc, via HK-dependent and -independent signal transduction, not only regulated root growth direction of vertically grown seedlings but also caused a significant drop in bending of roots under the stimulation of gravity in *Arabidopsis Thaliana* [33]. Starch is hydrolyzed into Glc during plant metabolism. AGPase is a key enzyme governing starch synthesis. In the study, HRW or Glc significantly enhanced the SS, SPS, HK, PK and AGPase enzyme activities. Conversely, GlcN significantly inhibited the enhancement caused by HRW (Figure 4). These results suggested that H_2_ promoted AR formation by enhancing the SS, SPS, HK, PK and AGPase enzyme activities. Kadowaki [34] reported that the activity of AGPase was also enhanced by sugar solution injections compared to the control, suggesting that the injection of sugar solutions is concluded to have a dual effect on root production in sweet potato. AGPase activity and production in roots was enhanced by injecting sucrose solution [35]. *SuSy1* played an important role in maintaining sucrose concentration in the cytoplasm of kiwifruit [9]. In *A. Thaliana*, AtHK1 accelerated senescence, enhanced the appearance of lateral buds and affected root growth [36]. *AtHK3* knockout mutant was found to be insensitive to 7% glucose, indicating that HK3 may play a role in sugar-sensing [37]. Transgenic rice plants overexpressing OsHXK5 or OsHXK6 exhibited growth inhibition and reduced expression of photosynthetic genes in response to glucose treatment [38]. We also found that HRW treatments were able to regulate higher expression levels of the sucrose metabolism-related genes including *CsSuSy1* and *CsSuSy6*, and glucose metabolism-related genes including *CsHK1*, *CsHK3*, *CsUDP1*, *CsUDP1-like*, *CsG6P1* and *CsG6P1-like* during AR formation (Figure 5). However, the upregulated gene expression was downregulated by GlcN (Figure 5). As mentioned above, H_2_ regulated AR formation by increasing the activities of SPS, SS, HK and PK enzymes and upregulating the expression levels of *CsSuSy1*, *CsSuSy6*, *CsHK1*, *CsHK3*, *CsUDP1*, *CsUDP1-like*, *CsG6P1* and *CsG6P1-like*.

## 4. Materials and Methods

### 4.1. Plant Material

The seeds of cucumber (*Cucumis sativus* L. ‘Xinchun NO. 4′) were soaked in 5% sodium hypochlorite solution for disinfection and immersed in distilled water for 6 h. The seeds were then transferred to a light incubator and maintained at 25 ± 1 °C for 6 days with a 14-h photoperiod at 200 μmol∙s^−1^∙m^−2^ intensity. The 6 day-old cucumber seedings whose primary roots were removed and used as explants were then placed in Petri dishes with distilled water or different chemicals indicated below under the same conditions of temperature and photoperiod described above for another 5 days. The treatment solutions are replaced every 8 h. Five days later, the number and length of AR per explant were measured and recorded. In addition, the explants were cultivated with different treatments under the same temperature and photoperiod conditions for 48 h. The stage belongs to the induction process without roots. Therefore, the hypocotyl base (1 cm) was collected and used for the following analysis. 

### 4.2. The Preparation of Hydrogen-Rich Water

Purified H_2_ (99.99%, *v*/*v*) was generated from a H_2_-producing apparatus (QL-300, Saikesaisi Hydrogen Energy Co., Ltd., Jinan, China). Firstly, prepared hydrogen gas was bubbled into 2 L distilled water (room temperature) at a rate of 300 mL·min^−1^. The processing was continued for 3 h. The prepared HRW was then analyzed by a dissolved hydrogen portable meter (ENH-1000, Trustlex Co., Led, Tokyo, Japan). The concentration of H_2_ was 0.45 mM, which was defined as 100% hydrogen rich water (HRW). Finally, HRW was immediately diluted to the required different concentrations (0.5%, 1%, 5%, 10%, 50% and 100%).

### 4.3. Explant Treatments

Cucumber explants were cultivated with various concentrations of Glc (0, 0.01, 0.05, 0.10, 0.50 and 1.00 mM). In addition, GlcN as the inhibitor of glucose was used in the study; refer to Woodward [39] research. The following chemicals were carried out: control (distilled water), 50% HRW, 0.10 mM Glc, 50% HRW + 0.10 mM Glc and 0.10 μM GlcN. Each process was set to three replicates. According to the initial experimental results, the concentration of the relevant chemical substance was determined. 

### 4.4. Determination Glucose Content

Glucose was analyzed as described by Abdellatif with some modifications [33]. Briefly, a 0.5 g sample was homogenized with 10 mL distilled water, diluted with distilled water into 50 mL volumetric flask and then mixed. The volumetric flask was then placed into a 50 °C water bath kettle for 10 min. Furthermore, 3,5-dinitrosalicylic acid (DNS) regent was prepared: 3,5-dinitrosalicylic acid (6.3 g) was dissolved in 262 mL 2 M sodium hydroxide solution. The hot-water solution of 500 mL containing 185 g potassium sodium tartrate was added. The two solutions were mixed until solvents were dissolved, followed by adding 5 g redistilled phenol and 5 g sodium sulfite, mixed and cooled to room temperature, and the volume adjusted to exactly 1000 mL with distilled water. The supernatant (2 mL) and 1.5 mL of DNS were mixed together. The mixture was centrifuged at 4000 r∙min^−1^ at 4 °C for 15 min. It was then placed in a boiling water bath for 5 min, cooled to room temperature, and then filtered into a 25 mL volumetric flask. The absorbance value was measured at 540 nm and the data were recorded. The relative content (compared to the control) is shown in Figure 2.

### 4.5. Determination Sucrose Content

The sucrose content was analyzed according to the procedure of Tauzin [40] making appropriate modifications according to experimental requirements. The sample (1.0 g) was homogenized with 10 mL of 80% ethanol and diluted with distilled water in a 100 mL volumetric flask. The volumetric flask was placed in a water bath at a constant temperature of 80 °C for 45 min, allowed to cool to room temperature and then filtered with a quantitative analysis filter paper with a diameter of 9 cm. The filter liquor was collected as the reaction liquid. The filter (0.4 mL) was mixed with 0.2 mL 2 M NaOH and bathed for 10 min at 80 °C; after cooling to room temperature, measurement of absorbance at 540 nm was carried out. The relative content (compared to the control) is shown in Figure 2.

### 4.6. Determination Starch Content

The method described by Smith was adopted to determine the starch content [41]. The frozen sample (0.5 g) was ground with 2 mL distilled water. Furthermore, 3.2 mL of 60% HClO4 was added. The solution was transferred to a 10 mL tube, diluted with distilled water to volume, and mixed. The solution was then centrifuged at 5000 r∙min^−1^ for 5 min, filtered and diluted with distilled water to 100 mL volumetric flask and mixed. The sucked supernatant (0.5 mL) was diluted to 3 mL with distilled water. Iodine reagent (2 mL) was added, mixed and left to stand for 5 min. Finally, it was diluted with distilled water to 10 mL. Distilled water was used as control and the absorbance was measured at 660 nm. The relative content (compared to the control) is shown in Figure 2.

### 4.7. Determination Total Sugar Content

Total sugar content was determined by procedures described according to Nath [42]. The 10 mL 6 M HCl was added to a 25 mL stopper tube to which 1 g sample was homogenized, and diluted to 25 mL with distilled water. The sugar was hydrolyzed during a 30 min water bath. Using phenolphthalein as its indicator, the total sugar content was determined with 6 M NaOH standard titration solution. Volume was then adjusted with distilled water to 100 mL, mixed and filtered. The filter liquor (10 mL) was transferred to a new test tube. It was then diluted to 100 mL with aqua distillate and analyzed. The absorbance value was measure at 540 nm and the data were recorded. The relative content (compared to the control) is shown in Figure 2.

### 4.8. Hexose Phosphate Content Measurements

Enzyme liquid was extracted by the modified method of Nägele and Wolfram [43]. In a nutshell, 0.5 g samples were ground in liquid nitrogen, then added to 2 mL 5% trichloroacetic acid (TCA) consisting of 100 mg PVPP. The mixtures were centrifuged at 12,000 r∙min^−1^ for 15 min at 4 °C. The supernatant was collected, and 150 μL neutralizing buffer containing 1 M triethanolamine and 5 M potassium hydroxide (KOH) was added to the supernatant. After a reaction for 30 min on ice, the mixture was then centrifuged at 12,000 r∙min^−1^ for 15 min. The supernatants were collected as the crude extract used for further analysis.

For G6P, F6P and glucose-1-phosphate (G1P), the reaction mixture consisted of 497 μL distilled water, 100 μL 1 M Hepes-KOH (pH 7.6), 100 μL 50 mM MgCl_2_, 100 μL 4 mM NAD, 100 μL 10 mM EDTA and 100 μL of the above crude extract. The formation of blue formazan was monitored by recording the absorbance at 560 nm. G6PDH, PGI and PGM were added successively for G6P, F6P and G1P mixtures, and then were determined by measuring the absorbance at 340 nm. The relative content (compared to the control) is shown in Figure 3.

### 4.9. SS, SPS, HK and PK AGPase Enzymes Activity Measurement

Enzyme extracts were performed as described previously [31]. Samples were harvested, ground in liquid nitrogen and homogenized in ice-cold 9 mL PBS (pH = 7.4). Extracts were then centrifuged at 3000 r∙min^−1^ for 15 min at 4 °C. The activities of sucrose synthase (SS), sucrose phosphate synthase (SPS), HK, pyruvate kinase (PK), and AGPase enzymes were measured by enzyme-linked immunosorbent assay (ELISA; AndyGene Biotechnology Co. Ltd., Beijing, China) according to the manufacturer’s instructions. The relative activity (compared to the control) is shown in Figure 4

### 4.10. Quantitative Real-Time PCR (qRT-PCR)

Total RNA was extracted using TRIzol (Invitrogen Life Technologies) by using the method described by Huang [44]. RNA was reverse transcribed with the 5 × *Evo M-MLV*RT Master Mix (AG, China) according to the manufacturer’s instructions. *Csactin* [35] was used as an internal control to calculate the relative expression. The relative transcript expression levels of the genes were quantified using 2^−ΔΔct^. The Cdna was amplified with 2 × SYBR Green *Pro Taq* HS Premix (AG, China) using the following primers shown in Table 2. The reactions were controlled by the following conditions: 30 s at 95 °C, then 5 s at 95 °C and 30 s at 60 °C for 40 cycles. 

### 4.11. Data Statistics and Analysis

Where indicated, results were expressed as the mean values ± SE of at least three independent experiments and 30 explants were taken for each replicate. Statistical analysis was performed using SPSS 22.0. For statistical analysis, Duncan’s multiple test (*p* < 0.05) was chosen as appropriate.

## 5. Conclusions

The present study provides new insights into the roles and interactions of H_2_ and Glc in the AR development in cucumber. It can be concluded that the H_2_ increased the glucose, sucrose, starch, total sugar, G6P, F6P and G1P contents during adventitious rooting. Meanwhile, the sucrose-related enzymes, including SS and SPS, and glucose-related enzymes, including HK, PK and AGPase activities, were increased by H_2_. Meanwhile, H_2_ also upregulated the expression levels of sucrose or glucose metabolism-related genes including *CsSuSy1*, *CsSuSy6*, *CsHK1*, *CsHK3*, *CsUDP1*, *CsUDP1-like*, *CsG6P1* and *CsG6P1-like*. In conclusion, Glc might be responsible for H_2_-regulated AR development.

## Figures and Tables

**Table 1:** Effect of Glc on AR development in cucumber.

Glc/mM	Root Number	Root Length (mm)
0.00	3.21 ± 0.18 c	4.99 ± 0.18 d
0.01	3.91 ± 0.12 c	6.97 ± 0.44 c
0.05	4.63 ± 0.14 b	8.04 ± 0.13 b
0.10	6.95 ± 0.05 a	9.69 ± 005 a
0.50	4.37 ± 0.01 b	7.06 ± 0.25 c
1.00	2.58 ± 0.18 d	4.06 ± 0.44 e

Effect of different concentrations Glc (0, 0.01, 0.05, 0.10, 0.50 and 1.00 mM) on AR development in cucumber explants. The values [mean ± standard error (SE)] are the average of three independent experiments (n = 30 explants per replicate). Values not sharing the same letters in the same list were significantly different by Duncan’s multiple-comparison test (*p* < 0.05).

**Table 2:** Sequences of primers used for RT-PCR analysis.

Gene Symbol	Accession Number ^a^	Primer Sequence (5′–3′)
*CsSuSy1-F*	LOC101213767	CGTGTGCTAAGGAAGGCGGAAG
*CsSuSy1-R*		CAGTGTCACCCCACCCTCTCTC
*CsSuSy6-F*	LOC101216865	TCCAACCGCCACAACTTCATCAC
*CsSuSy6-R*		CCATTCCCACTCTGCCCAAGC
*CsHK1-F*	LOC101218300	CGCCATGACCGTCGAGATGC
*CsHK1-R*		TTTGTACCGCCGAGATCCAATGC
*CsHK3-F*	LOC101215511	CACGGTCCTAGTCAGTCGGAGAG
*CsHK3-R*		GCCATAGCATCAACCACCTGTCTC
*CsUDP1-F*	LOC101206505	TCCAGAGTTCCTTGCTGAGGGTAC
*CsUDP1-R*		AAGCCTGAATTGCCTTGAGACCATC
*CsUDP1-like-F*	LOC116401645	AGTTAATGCCATTTCCGCCCTCTG
*CsUDP1-like-R*		TCTTGTATCCGTACCAACCGAATGC
*CsG6P1-F*	LOC101222586	AGGTGCGATTGCTAATCCAGATGAG
*CsG6P1-R*		TGCGACTTCAAGAACGAGTTAGGTG
*CsG6P1-like-F*	LOC101210696	AGGGTGGAGGTTTAGGGTTTAGGG
*CsG6P1-like-R*		GCCGCTCGTTCATTCCATTGTTC

^a^ NCBI database

## Data Availability

All data, tables and figures in this manuscript are original.
